# Item-specific delay activity demonstrates concurrent storage of multiple active neural representations in working memory

**DOI:** 10.1371/journal.pbio.3000239

**Published:** 2019-04-26

**Authors:** David W. Sutterer, Joshua J. Foster, Kirsten C. S. Adam, Edward K. Vogel, Edward Awh

**Affiliations:** Department of Psychology and Institute for Mind and Biology, University of Chicago, Chicago, Illinois; Vanderbilt University, UNITED STATES

## Abstract

Persistent neural activity that encodes online mental representations plays a central role in working memory (WM). However, there has been debate regarding the number of items that can be concurrently represented in this active neural state, which is often called the “focus of attention.” Some models propose a strict single-item limit, such that just 1 item can be neurally active at once while other items are relegated to an activity-silent state. Although past studies have decoded multiple items stored in WM, these studies cannot rule out a switching account in which only a single item is actively represented at a time. Here, we directly tested whether multiple representations can be held concurrently in an active state. We tracked spatial representations in WM using alpha-band (8–12 Hz) activity, which encodes spatial positions held in WM. Human observers remembered 1 or 2 positions over a short delay while we recorded electroencephalography (EEG) data. Using a spatial encoding model, we reconstructed active stimulus-specific representations (channel-tuning functions [CTFs]) from the scalp distribution of alpha-band power. Consistent with past work, we found that the selectivity of spatial CTFs was lower when 2 items were stored than when 1 item was stored. Critically, data-driven simulations revealed that the selectivity of spatial representations in the two-item condition could not be explained by models that propose that only a single item can exist in an active state at once. Thus, our findings demonstrate that multiple items can be concurrently represented in an active neural state.

## Abstract 2

An electroencephalography-based study shows that working memory delay activity concurrently represents two items, posing a challenge for models which propose that only one item can be actively represented at a time.

## Introduction

Working memory (WM) is an “online” memory system that maintains information in a readily accessible state. It has long been thought that persistent, stimulus-specific activity plays a central role in the maintenance of information in WM [1]. This view is supported by considerable evidence that visual features maintained in WM can be decoded from patterns of persistent neural activity in humans and non-human primates alike [2–5]. Although there is broad agreement that persistent activity is central to maintenance in WM, there is debate regarding how many items can be represented in an active state (i.e., represented by persistent neural activity) at once.

On the one hand, some researchers have embraced a strict one-item limit on the number of items that can be represented “online.” For example, McElree and colleagues [6,7] proposed a two-state model of memory, comprising a single-item focus of attention, with all other information relegated to a passive state in long-term memory. Taken together with other work that has demonstrated links between the current focus of attention and the presence of decodable neural activity [8–13], there is motivation for the hypothesis that there may be a strict—perhaps single-item—limit on the number of neurally active representations.

On the other hand, other researchers have argued that load-dependent neural activity provides evidence for the concurrent maintenance of multiple active representations in WM. For example, sustained electroencephalography (EEG) [14] and functional Magnetic Resonance Imaging (fMRI) [15] signals track the number of items maintained in WM, reaching a plateau at set sizes consistent with past estimates of item limits in visual WM [16–19]. However, load-dependent signals do not provide unequivocal evidence for concurrently active neural representations because they could also track other processes that change with memory load. For example, a strict one-item limit could require that multiple items are switched in and out of an active state when more than 1 item is behaviorally relevant. Switching frequency might increase with the number of relevant items, providing a possible explanation for activity that scales with memory load when only 1 item is active at a time.

Stimulus-specific neural activity may provide more traction on this question. Past work has found evidence for stimulus-specific delay activity for multiple items held in WM [20–25]. However, this work does not firmly establish that multiple items are concurrently stored in an active state. Studies that have manipulated memory load have found that the selectivity of stimulus-specific delay-period activity declines with increasing memory load [20,21,23–25]. The reduced selectivity of neural representations with greater memory load might occur because competition between concurrently stored representations degrades the fidelity of those representations [26,27]. However, if only 1 item can be actively represented at once, each item may transition between active and silent states, with only a single item in an active state at any given time. For example, Lundqvist and colleagues [28,29] have argued for a model in which bursts of activity represent the items maintained in WM, such that information is represented in an activity-silent state between these bursts (also see [13,30–32]). Furthermore, other work suggests that when 2 locations must be attended, these locations are sampled rhythmically at a rate of 4 to 7 Hz [33–38]. Given past work positing a functional overlap between spatial WM and spatial attention [39–41], sequential representation may also underpin the maintenance of multiple locations in spatial WM. Such a “switching model,” in which only 1 item is actively represented at once, also predicts an apparent decline in the fidelity of neural representations when mnemonic load is increased, because typical analyses aggregate data across multiple trials (e.g., [21,24]).

Here, we directly tested whether persistent neural activity can concurrently represent multiple items. Human observers maintained the locations of 1 or 2 items over a brief delay period. We tested whether multiple locations are concurrently represented in oscillatory alpha-band (8–12 Hz) activity, which—past work has shown—precisely encodes spatial locations maintained in WM [42,43]. To this end, we used an inverted encoding model (IEM) [44–46] to reconstruct representations of the remembered locations from the scalp distribution of EEG alpha-band power [42,43]. Consistent with past work, we found that the selectivity of stimulus-specific activity was reduced when 2 items were remembered than when 1 item was remembered. Critically, however, when we simulated the expected selectivity of stimulus-specific activity during the two-item condition assuming a switching model (in which only 1 item was actively represented at once), we found that the observed selectivity in the 2-item condition was reliably higher than that predicted by a switching model. Thus, our findings provide evidence that multiple items can be concurrently represented in a neurally active state.

## Results

### Behavior

**Fig 1:**
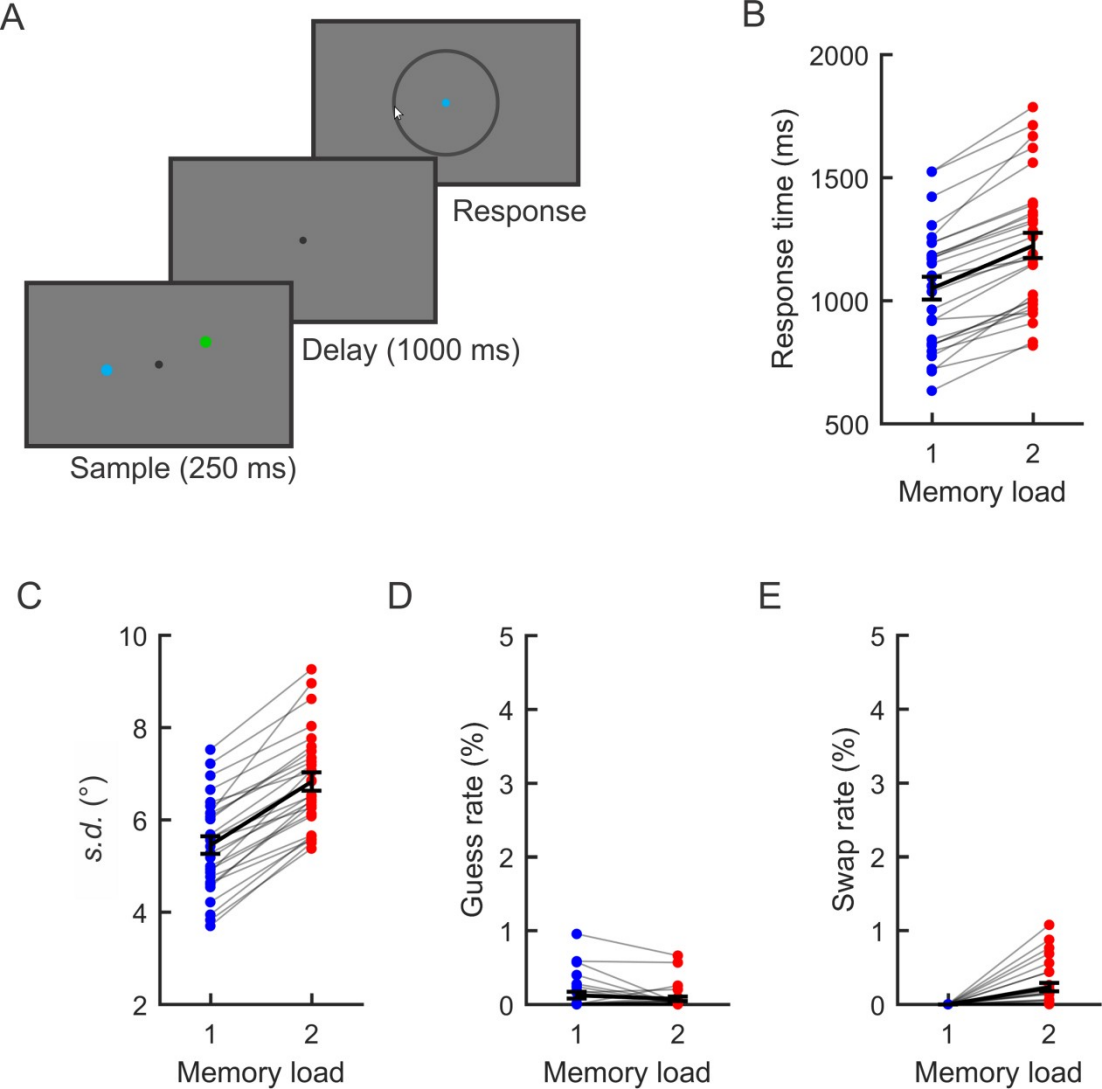
Experimental task and behavior. (A) Observers saw a brief sample display (250 ms) that contained 1 or 2 colored dots. After a delay period (1,000 ms), the fixation point changed color (blue or green) to indicate which item should be reported. Observers reported the angular position of the cued item as precisely as possible by mouse click on the perimeter of a rim. (B) Median response time (ms) as a function of memory set size (1 versus 2 items). Light grey lines represent individual observers. Black lines represent the mean. Error bars represent ±1 SEM. (C–E) Parameter estimates obtained by fitting a three-component mixture model [50] to response errors as a function of memory set size. *s*.*d*. (panel C) reflects precision of responses (with higher values indicating worse precision), *pGuess* (panel D) estimates the probability that the observer produced a random response (i.e., a guess), and *pSwap* (panel E) estimates the probability that the uncued item was misreported instead of the cued item. Note that *pSwap* is necessarily 0 for the single-item condition. Data are available at https://osf.io/47cmn/. *s*.*d*., standard deviation of von Mises distribution.

Observers performed a spatial WM task (Fig 1A). On each trial, observers remembered the spatial position of 1 or 2 colored dots and reported the position of a cued item with a mouse click. We found that median response times (Fig 1B) were slower for two-item trials (*M* = 1,224; standard deviation [SD] = 271) than one-item trials (*M* = 1,050 ms; SD = 245) (*t*[27] = 12.33; *p* < 0.0001). In line with past work [47–50], memory performance declined as memory load increased from 1 to 2 items. We analyzed the recall data using a three-component mixture model [50] to estimate mnemonic precision (standard deviation of von Mises distribution [*s*.*d*.]; higher values indicate lower precision), the probability that a stimulus was forgotten (*pGuess*), and the probability of reporting the non-target item (*pSwap*). We found that mnemonic precision was worse (i.e., *s*.*d*. was higher [Fig 1C]) when participants maintained 2 items (*M* = 6.83°, SD *=* 1.05) than when they maintained 1 item (*M* = 5.45°, SD *=* 1.01) (*t*[27] *=* 15.66; *p* < 0.0001). We saw no reliable difference in the rate of guessing (Fig 1D) between one-item (*M* = 0.12%, SD = 0.24) and two-item (*M* = 0.08%, SD = 0.16) trials (*t*[27] = −1.60; *p* = 0.121). Finally, observers’ rates of misreporting the location of the non-target item (Fig 1E) on two-item trials were low (*M =* 0.23%, SD *=* 0.30). Note that the combined rate of guessing and swapping were very low (less than 1% for almost all observers). Thus, the primary changes in behavior with memory load were the slowing of response times and reduction in precision of responses.

Responses were very precise in the two-item condition. However, it is possible that when the 2 items were close together, observers may have remembered just 1 location. For example, observers may have remembered a location between the items, or randomly remembered the location of just 1 of the items. To test this possibility, we further analyzed two-item trials in which the items occupied the same position bin (i.e., the same 45° wedge of positions). For these trials, we calculated response error relative to the “unprobed item,” in addition to response error relative to the probed item. If observers remembered just 1 location on these trials rather than remembering the precise position of both of the items, we reasoned that response error relative to the probed and unprobed items should be equivalent. Instead, we found that response error was considerably smaller relative to the probed item (*M* = 4.54°, SD = 0.80) than relative to the unprobed item (*M* = 19.28°, SD = 2.11) (*t*[27] = 42.70; *p* < 0.0001). This was also the case when we restricted the analysis to trials in which the 2 items were less than 10 degrees (angular position) apart: responses were more tightly clustered around the probed item (*M* = 4.29°, SD = 1.56) than the unprobed item (*M* = 9.39°, SD = 2.23) (*t*[27] = 17.78, *p* < 0.0001). Thus, observers precisely remembered both spatial positions when the 2 items were close to each other.

### Alpha-band representations of space degrade with increased memory load

**Fig 2:**
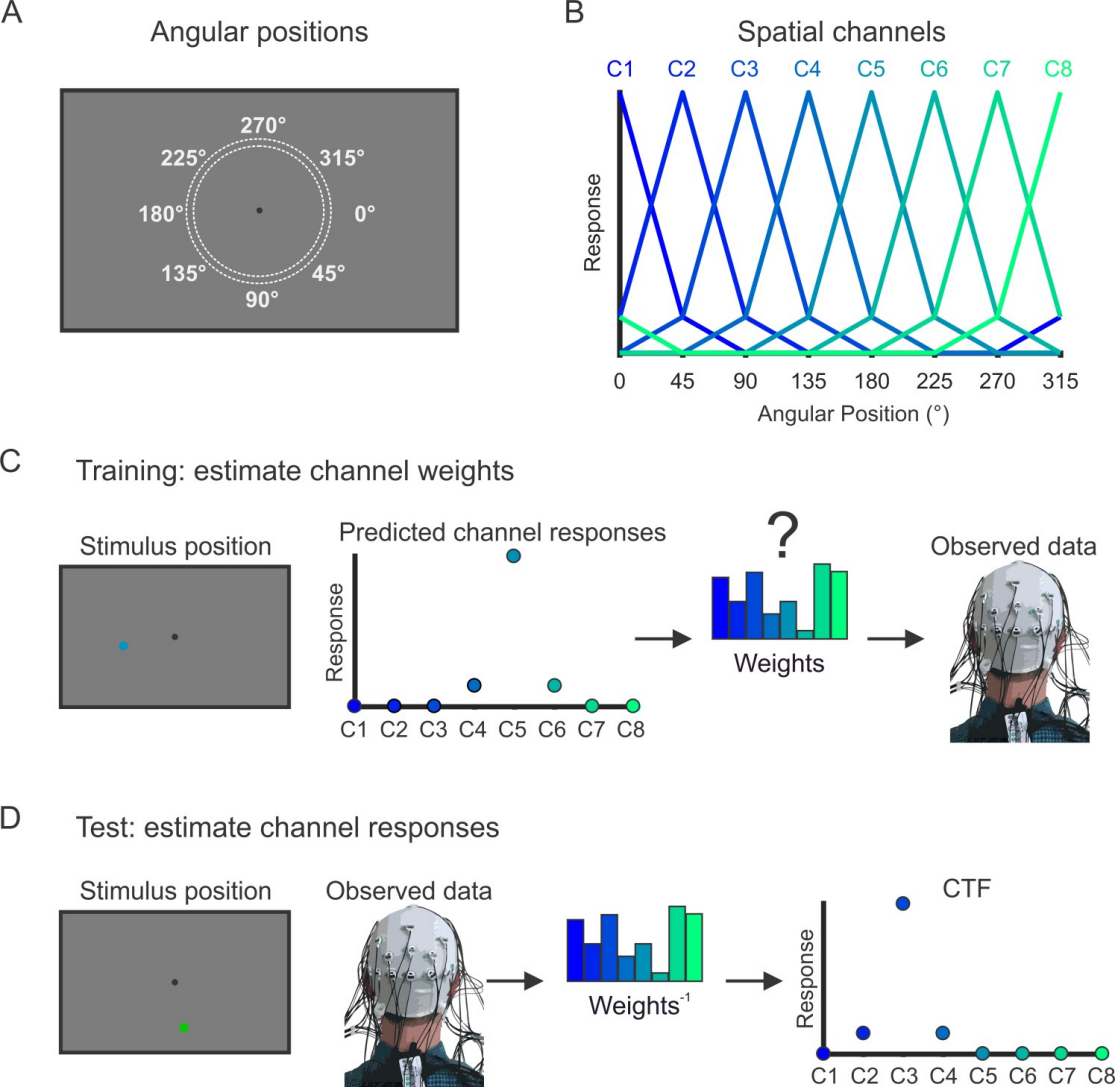
IEM procedure for reconstruction spatial CTFs. (A) The stimuli varied in angular position around the fixation point. Stimuli were categorized as belonging to 1 of 8 positions bins (a 45° wedge of positions), centered at 0°, 45°, and so forth. (B) We modeled power at each electrode as a linear combination of the 8 hypothetical spatial channels (C1–C8). The curves show the predicted response of the spatial channels across angular positions. (C) In the training phase of the analysis, we estimated the relative contributions of each of the 8 channels to power measured at each electrode (called “channel weights”). (D) In the test phase of the analysis, we used the channel weights obtained during training to estimate the channel responses from the multivariate pattern of power across electrodes. Critically, the data used in the training and test phases of the analysis were independent to avoid circularity. For details, see Materials and methods. Data are available at https://osf.io/47cmn/. CTF, channel-tuning function; IEM, inverted encoding model.

To test how online representations change with increased memory load, we examined oscillatory alpha-band (8–12 Hz) activity, which encodes spatial representations that are maintained in WM [42,43]. We used an IEM [44–46] to reconstruct spatial representations encoded by alpha-band activity [42,43]. Our encoding model assumes that alpha-band power measured at each scalp electrode reflects the activity of a number of spatially tuned channels (or neuronal populations), each tuned for a different position in the visual field (Fig 2A and 2B). In a training phase, we estimated the relative contributions of the spatial channels to each electrode on the scalp (called the “channel weights”) using a subset of trials during the spatial WM task (Fig 2C). Then, in a test phase, using an independent subset of trials, we used these channel weights to estimate the responses of the spatial channels given the pattern of alpha-band power across the scalp (Fig 2D). The resulting profile of responses across the spatial channels (called channel-tuning functions [CTFs]) reflects the spatial selectivity of alpha-band activity measured by EEG. We performed this analysis at each time point throughout the trial, which allowed us to test whether active spatial representations were maintained throughout the delay period.

**Fig 3:**
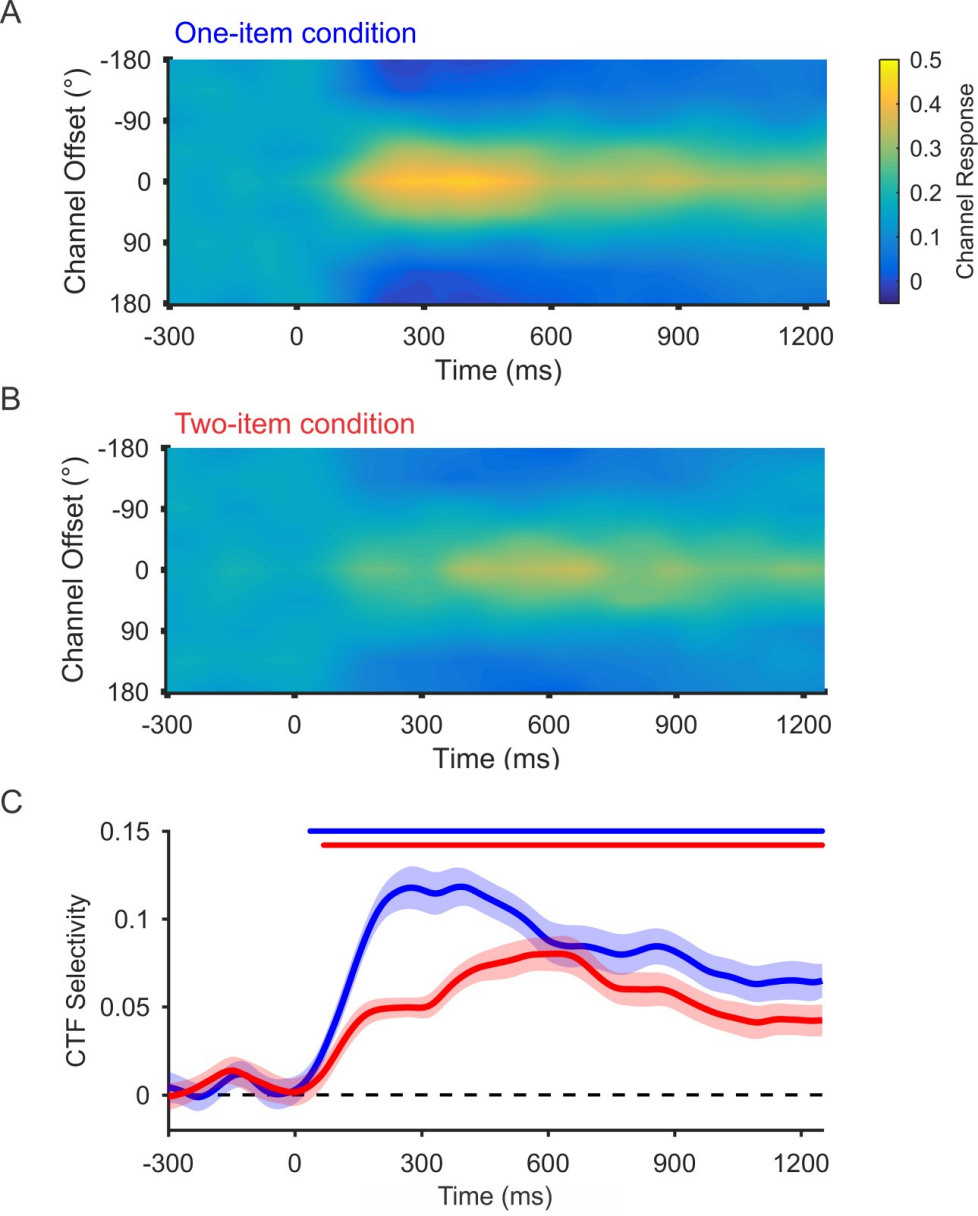
Spatial alpha-band CTFs as a function of memory load. Average alpha-band CTF in the one- and two-item conditions (A and B, respectively). (C) The spatial selectivity of alpha-band CTFs across time (measured as CTF slope, see Materials and methods) as a function of memory load. The blue (one-item) and red (two-item) markers at the top of the panel indicate the period of above-chance selectivity obtained using a cluster-based test. CTF selectivity was reliably higher in the one-item condition than in the two-item condition. The shaded error bars reflect ±1 SEM across observers. Data are available at https://osf.io/47cmn/. CTF, channel-tuning function.

To examine how WM load affects alpha-band representations of the remembered positions, we reconstructed CTFs for the both the one- and two-item conditions (having estimated channel weights using a training set that included data from both the one-item and two-item conditions; see Materials and methods). Note that we reconstructed CTFs for the probed item in both conditions. In the two-item condition, this choice was arbitrary because both items needed to be prioritized on the two-item trials. Thus, the 2 items (probed and unprobed) are no different in status until after the delay period. In this analysis, we were able to isolate the CTF for the probed item from that of the unprobed item because the position of the unprobed item was independent of the probed item (i.e., the location bins that the 2 items occupied were fully counterbalanced). Thus, no spatially selective activity corresponding to the unprobed item will be seen when the channel responses are aligned to the position of the probed item. We observed a clear spatially selective CTF, with a peak response in the channel tuned for the remembered location of the probed item (a channel offset of 0°), which persisted throughout the delay period in both the one-item and two-item conditions (Fig 3A and 3B). Fig 3C shows the spatial selectivity of the CTFs seen in each condition across time (measured as CTF slope; see Materials and methods). Cluster-based permutation tests revealed that spatial selectivity of alpha-band CTFs was reliably above 0 throughout the delay period for both the one- and two-item conditions (*p <* 0.05, corrected for multiple comparisons; see markers at the top of Fig 3C). Next, we compared CTF selectivity for the one-item and two-item conditions. Delay-period CTF selectivity (averaged from 250 to 1,250 ms after stimulus onset) was reliably lower for two-item trials (*M* = 0.059, SD = 0.033) than for one-item trials (*M* = 0.087, SD = 0.046) (*t*[27] = 4.86; *p* < 0.0001). We also found that this difference was reliable during a late window (800–1,250 ms) when alpha-band representations are unlikely to be affected by stimulus-driven activity (*t*[27] = 2.99; *p* = 0.006). Thus, as memory load increases, there is a decline in spatially selective alpha-band activity that tracks the stored locations.

### Ruling out methodological explanations for the load-related decline in CTF selectivity

As outlined above, we isolated the CTF for the probed item from that of the unprobed item because the positions of the 2 items were independent (i.e., the location bin that the 2 items occupied were fully counterbalanced). Because the position of the unprobed item was random relative to the probed item, the representation of the unprobed item will function as noise when reconstructing the probed item, which might interfere with the reconstruction of the probed item. Thus, we considered whether the reduced CTF selectivity seen in the two-item condition could reflect an inability of our IEM procedure to accurately reconstruct the representation of interest when a second representations is present, rather than a true decrease in the spatial selectivity of neural representations with greater memory load. To test this possibility, we applied our IEM analysis to simulated data in which we varied the number of representations (1 versus 2) while holding the selectivity of the representations constant (for details, see Materials and methods, “Encoding model simulations”). When we held selectivity constant across the 2 conditions in the simulated data, we found that adding a second representation did not affect the selectivity of the reconstructed CTFs (S1A Fig).

More generally, there could be other sources of noise in the measured neural activity that vary systematically with memory load, which might also degrade reconstructed CTFs. In a second simulation, we tested whether greater noise in the two-item condition can explain the decline in CTF selectivity with memory load. In this simulation, we added more Gaussian noise to simulated data in the two-item condition than to the one-item condition. We found that mean CTF selectivity still did not reliably differ between the 2 conditions. However, we did find that the estimated CTF selectivity was more variable in the higher-noise two-item condition, such that noisier data produce noisier CTFs but do not systematically bias CTF selectivity (S1B Fig).

Finally, in a third simulation, we reduced the amplitude of the spatial selectivity in the simulated two-item data compared to the simulated one-item data (holding noise constant between the conditions). Here, we found that the selectivity of the reconstructed CTFs was lower for the two-item condition than the one-item condition (S1C Fig), confirming that the IEM procedure is sensitive to changes in selectivity of spatial representations. Taken together, these simulations suggest that the drop in CTF selectivity that we observed as memory load increased cannot be explained by (1) the second representation interfering with our ability to reconstruct the probed item or by (2) greater noise in the two-item condition than in the one-item condition. Thus, we can be confident that the drop in CTF selectivity that we observed reflects a true drop in the selectivity of spatial representations held in WM.

### Spatial alpha-band representations have a similar format in one-item and two-item conditions

**Fig 4:**
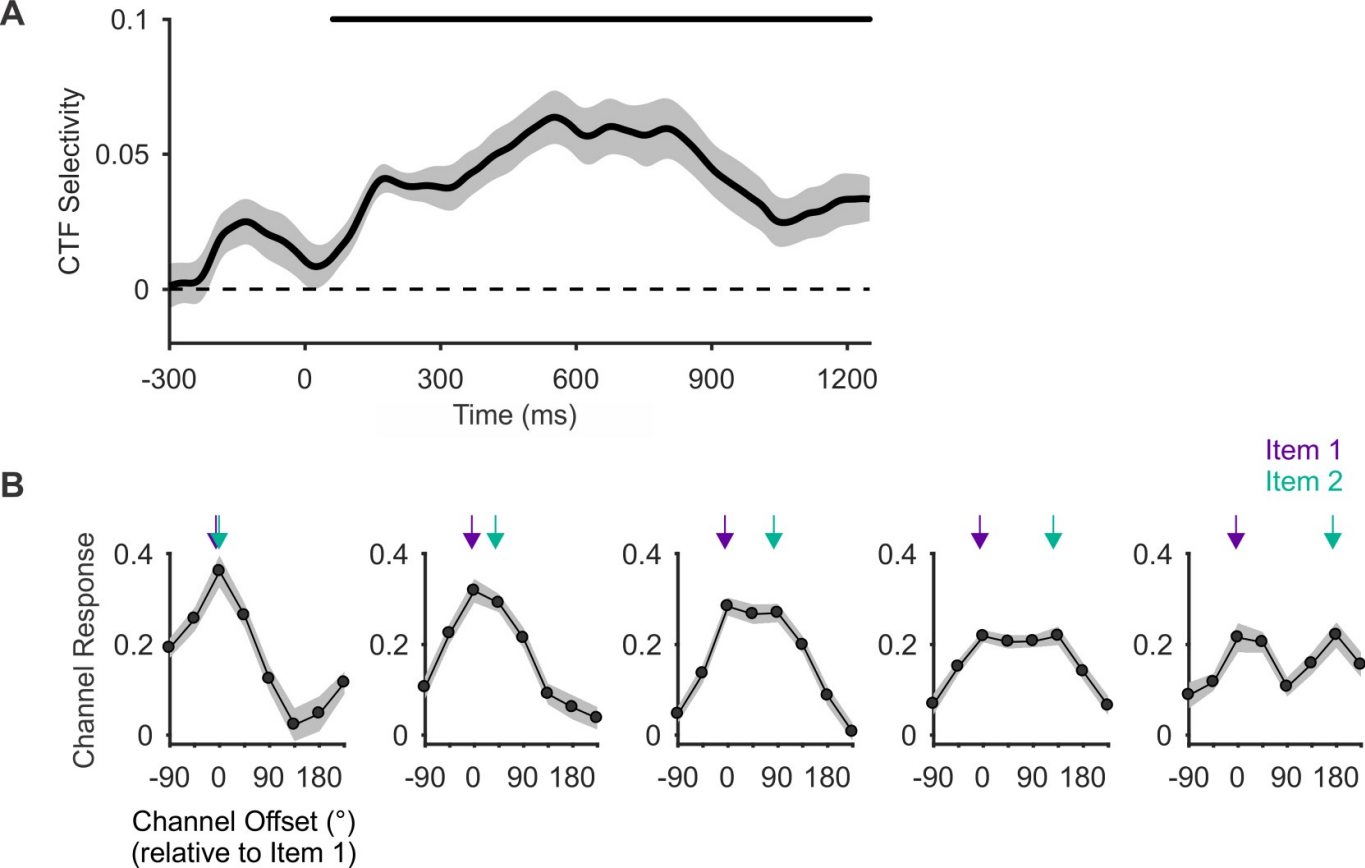
Testing whether multivariate patterns of alpha-band power generalize between one-item and two-item trials. (A) The spatial selectivity of alpha-band CTFs across time (measured as CTF slope; see Materials and methods) in the two-item condition when the IEM was estimated using the one-item condition. The markers at the top of the panel indicate the period of above-chance selectivity obtained using a cluster-based test. (B) Mean delay-period (averaged from 250–1,250 ms) channel-response profiles as a function of the distance between the items. The arrows above each subplot mark the channel(s) tuned for the positions of the 2 stimuli. The shaded error bars reflect ±1 SEM across observers. Data are available at https://osf.io/47cmn/. CTF, channel-tuning function; IEM, inverted encoding model.

Our analysis comparing CTF selectivity across the one-item and two-item conditions assumed that the pattern of alpha-band activity that corresponds to each remembered location is not fundamentally different when 1 or 2 items are remembered, as could be the case if observers recoded the locations in some way in the two-item condition (e.g., recoded as a single configuration or chunk). To test this assumption, we performed a cross-training analysis, in which we trained the IEM on the one-item condition and tested on two-item condition. This analysis revealed robust reconstructions of the location of the probed item in the two-item condition (Fig 4A). Therefore, estimating the IEM based on one-item data enabled accurate reconstruction of spatial representations in the two-item condition. Next, we ran a second cross-training analysis in order to visualize the representations for both items at once. To this end, we separately reconstructed channel responses for trials in which the 2 items were separated by a fixed distance. Fig 4B shows average delay-period channel-response profiles (250–1,250 ms) as a function of the distance between the 2 items. When the 2 items were 180° apart, we observed 2 clear peaks in the channel-response profile, corresponding to the location of each item. When items were closer together, these 2 peaks were superimposed in an orderly manner. Thus, the pattern of alpha topography in the two-item condition is well described as a superimposition of the patterns that would be expected for each item by itself. Although this analysis cannot rule out all possible differences in format that could have arisen as mnemonic load increased from 1 to 2 items, these findings establish a strong level of correspondence between the alpha topographies in the 2 load conditions.

### Alpha-band activity concurrently encodes 2 spatial representations

**Fig 5:**
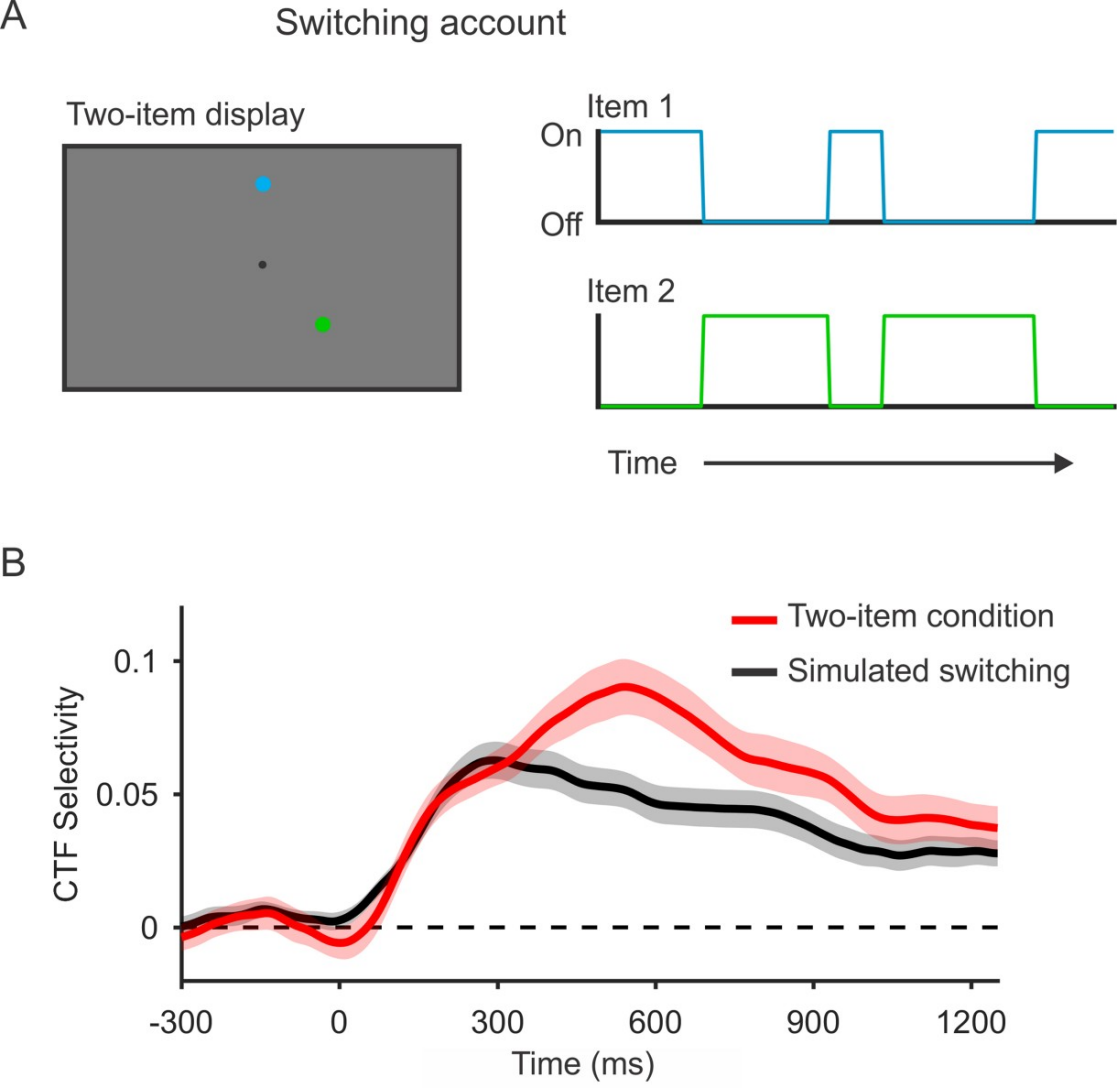
Alpha-band activity concurrently represents 2 spatial positions. (A) If only 1 item can be represented by alpha-band activity at a time, then in the two-item condition, the items might alternate between an active and an activity-silent state such that only 1 item is actively represented at once. We used data from the single-item condition to simulate the expected CTF based on this switching account. This account holds that each item is represented 50% of the time (on average). Thus, we simulated switching between items by randomizing the position labels for 50% of trials for the one-item condition. (B) Spatial selectivity of alpha-band CTFs across time (measured as CTF slope) for the two-item condition (red) and for simulated switching (black). CTF selectivity was reliably higher during the two-item condition (red) than for simulated switching (black). The shaded error bars reflect ±1 SEM across observers. Data are available at https://osf.io/47cmn/. CTF, channel-tuning function.

Consistent with past work [20,21,24,25], we observed that spatially specific alpha-band activity deteriorates as memory load increases. Such declines in stimulus-specific activity with increasing load have typically been attributed to a loss of memory fidelity due to competition between multiple active representations [26,27]. However, another possibility is that only a single item is maintained in an active state at any given moment, while the other items stored in WM are represented in an activity-silent state [6,11,12,31]. Under this account, the apparent persistent activity in the two-item condition (Fig 3B) can be explained if the 2 items in WM switch in and out of an active state (Fig 5A). This switching account asserts that with a memory load of 2 items, each item can only be represented a maximum of 50% of the time, on average. To test whether this switching account can explain the CTFs seen during the two-item condition, we simulated the CTF selectivity expected under this account. We reasoned that if the switching account is true, then CTF selectivity for each item, when it is actively represented, should be equivalent to that seen in the one-item condition because only 1 item is actively represented at a time. Therefore, we generated CTFs from the single-item condition but randomized the position labels for 50% of the trials. We then compared CTF selectivity seen during the two-item condition with the CTF selectivity expected under the switching account. If the switching account was correct, we should see no difference between CTF selectivity for two-item trials and for the simulated switching conditions. However, if we observed a higher CTF selectivity for the observed two-item data than for simulated switching, we could conclude that alpha activity must simultaneously encode multiple locations in WM during a given trial.

Fig 5B shows CTF selectivity across time for the two-item condition and for simulated switching based on the one-item data. We found that CTF selectivity was higher throughout the delay period (averaged from 250 to 1,250 ms after stimulus onset) for the two-item condition (*M* = 0.063, SD = 0.036) than expected based on the switching account (*M* = 0.043, SD = 0.030) (*t*[27] = 5.27; *p* < 0.0001). We also observed a reliable difference when we restricted our analysis to a window late in the delay period (800–1,250 ms) to minimize the contribution of stimulus-driven activity (*t*[27] = 2.87; *p* = 0.008). This analysis provides evidence that the spatial positions of both items were simultaneously represented by alpha-band activity. We note that this finding does not established that observers always represent both items in a neurally active state. Instead, it is possible there are periods during which only 1 of the 2 items are actively represented. Thus, while our findings demonstrate the simultaneous representation of 2 distinct positions in spatially selective alpha activity, they do not establish that 2 items were continuously represented without interruption.

### The frequency of oscillations that encode spatial representations does not change with memory load

**Fig 6:**
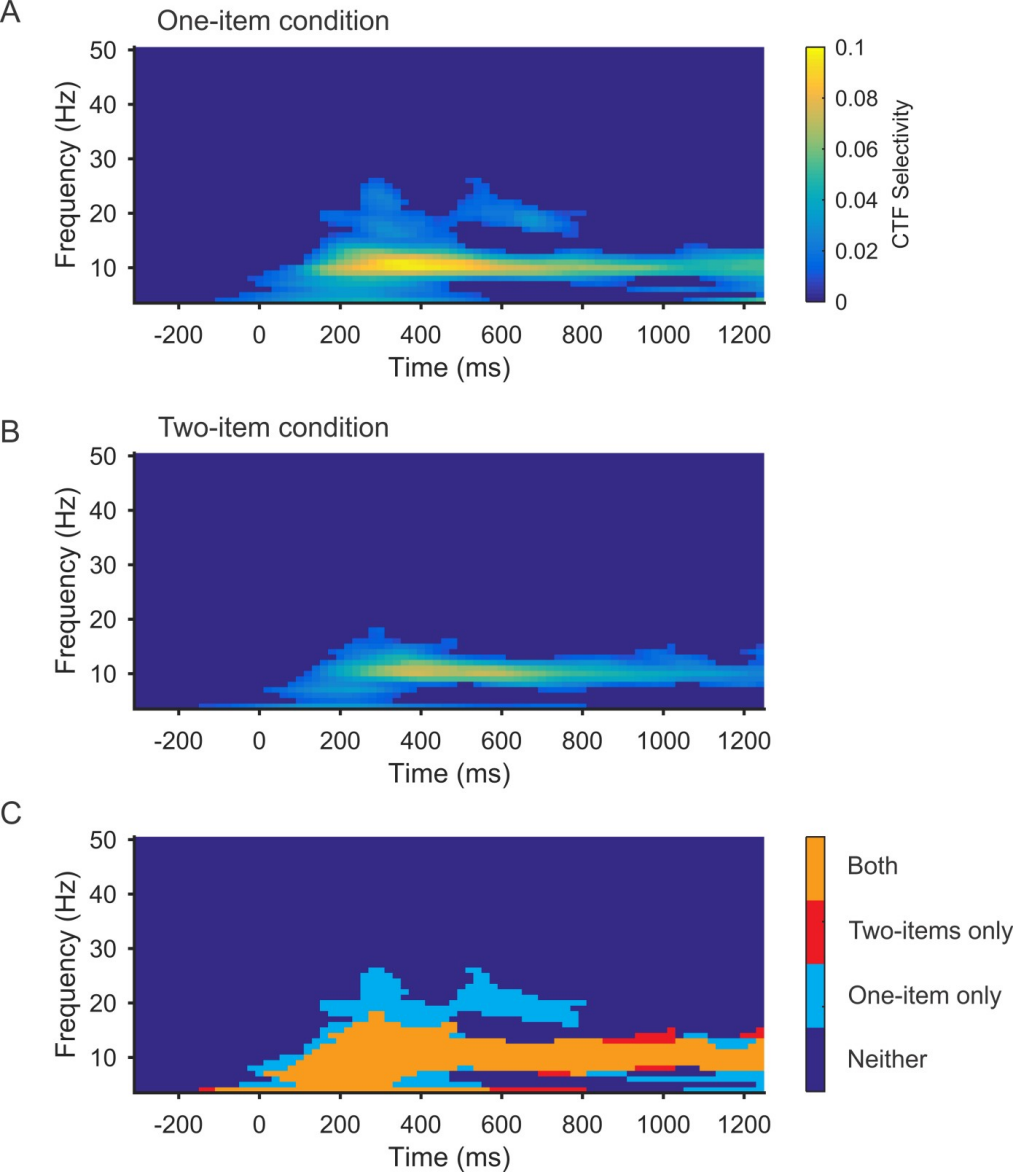
Identifying frequencies that track remembered positions during one-item and two-item maintenance. (A–B) Selectivity of spatial CTFs (measured as CTF slope) reconstructed from the scalp distribution of oscillatory power across a broad range of frequencies for the one-item (A) and two-item (B) conditions. Points with no reliable CTF selectivity as determined by the cluster-corrected permutation test are set to dark blue. (C) Overlay plot marking the clusters of reliable selectivity in the one-item condition (light blue), two-item condition (red), and both (orange). Data are available at https://osf.io/47cmn/. CTF, channel-tuning function.

The decline in the spatial selectivity of alpha-band activity with increasing memory load suggests that the fidelity of the spatial representations decreased as memory load increased. However, another possibility is that the remembered locations were represented by a different frequency band when memory load increased. To test this possibility, we performed the IEM analysis separately for the one-item and two-item conditions (i.e., both training and testing within each condition; see Materials and methods) across a range of frequencies (4–50 Hz). We conducted a cluster-corrected permutation test to identify reliable clusters of above-chance CTF selectivity. Consistent with our past work [42], just after sample onset we observed a transient burst of spatially specific activity across a range of frequencies (4–25 Hz). However, only alpha-band activity (8–12 Hz) tracked the remembered position(s) throughout the delay period (Fig 6A and 6B). An overlay plot of spatially specific frequencies in both one-item and two-item trials revealed a similar frequency profile later during the delay period, when stimulus-driven activity has subsided (Fig 6C). These findings show that the frequency of oscillatory activity that encodes spatial representations does not change with memory load. Thus, the decrease observed in the spatial selectivity of alpha-band CTFs with increasing memory load reflects a decline in spatially selective activity rather than a shift in the frequency of spatially selective oscillations.

## Discussion

A longstanding view is that persistent neural activity plays a central role in the maintenance of information in WM. In support of this view, past work has shown that stimulus-specific patterns of delay-period activity track visual features maintained in WM [1–5]. However, there has been considerable debate regarding the number of items that can be represented in an active state (i.e., coded by persistent neural activity) at any given moment, with estimates varying from as few as just 1 item [6,11], to as many as 3–4 items [17–19]. Although past work has found that multiple representations held in WM can be decoded from delay activity [20–22,24,25], these results can be explained by models that propose that only a single item is actively represented at a time. According to this view, the apparent representation of multiple items is seen because typical analyses aggregate data across trials, averaging together periods when an item is actively represented and periods when it is not. Indeed, this view readily accounts for the finding that the selectivity of stimulus-specific activity declines with increasing memory load [20,21,24]. If only 1 item is represented in an active state at once, and items are switched in an out of this state, then this would produce a decline in stimulus-specific activity when averaging across trials.

Here, we tested this switching account, in which only 1 item can be actively represented at once. We used an IEM combined with EEG measurements of oscillatory alpha-band (8–12 Hz) power to track remembered spatial locations during a WM task in which human observers remembered 1 or 2 spatial locations. Consistent with past work that has found that stimulus-specific activity declines with WM load [20,21,24], we found that the spatial selectivity of alpha-band activity declined as memory load increased. We tested the switching account by simulating selectivity expected under a switching model in which only 1 item was actively represented at a time. We found that the spatial selectivity of alpha-band CTFs during two-item trials was greater than expected if only 1 item was actively represented at a time. Thus, our findings show that multiple items can be represented concurrently in an active neural state.

In principle, our switching simulation can also be applied to existing fMRI studies that have examined stimulus-specific activity as a function of memory load [21,24,25]. However, the blood oxygen level–dependent (BOLD) signal measures the hemodynamic response as a proxy for neural activity rather than measuring neural activity directly. Thus, given the slow time course of the hemodynamic response, evidence for concurrent representations in the BOLD signal does not necessarily indicate concurrent representations in neural activity. For example, if 2 stimuli are rapidly represented in sequence (i.e., not concurrently), both stimuli will nevertheless be concurrently represented in the BOLD signal because a transient burst of neural activity produces a sustained BOLD response, peaking 4 to 6 seconds after the burst of activity. In contrast, alpha-band activity is a direct measure of electrical brain activity, allowing us to safely conclude that both items were concurrently represented by neural activity.

### Assumptions

Our approach to testing whether multiple items are concurrently represented in an active state relies on 3 assumptions. First, we assumed that the frequencies and patterns of activity that encode spatial position do not vary with memory load. In support of this assumption, we found that spatial representations are encoded by oscillatory activity in the alpha frequency band in both the one- and two-item conditions (Fig 6). We also found that training the encoding model using data from the one-item condition enabled successful reconstruction of the remembered position in the two-item condition, providing evidence that the patterns of activity that encode spatial position do not vary with memory load. However, no known analysis can completely rule out the possibility that there are differences—opaque to the method or analysis—in how patterns of activity encode spatial position between one- and two-item trials. That said, when comparing the spatial selectivity of one- and two-item alpha-band representations, we estimated the encoding model with data from both conditions and then reconstructed CTFs for each condition separately. A feature of this approach is that if there are differences between how spatial positions are represented in the 2 conditions, then CTF selectivity should be underestimated in both conditions because our training set, which includes data from both conditions, would be suboptimal for both conditions. Thus, we think it is unlikely that slight differences in how spatial position is encoded between the conditions can explain our core result that CTF selectivity in the two-item condition is greater than would be expected by a switching account.

Second, we assumed that the time spent off task (i.e., not representing the to-be-remembered stimuli) is not greater in the one-item than in the two-item condition. If this assumption was violated, such that observers spent more time off task in the one-item condition than in the two-item condition, our expected CTF selectivity under a switching account would have been too low. Thus, CTF selectivity in the two-item condition could have exceeded selectivity expected under the switching account not because observers maintain concurrent representations in the two-item condition, but because observers spent more time off task in the one-item condition than in the two-item condition. Recent work provides some evidence against this possibility. Unsworth and Robison found that self-reported mind wandering—which predicts lapses in task performance [51,52]—did not vary with memory load in a visual WM task [53]. Our task was similar to Unsworth and Robison’s in that memory load was also randomly interleaved (not blocked), and our procedure involved similar timing (250 ms encoding; 1,000 ms delay versus 250 ms encoding; 900 ms delay). Therefore, it seems reasonable to assume that there were similar levels of mind wandering across the one-item and two-item conditions in the present study as well. That said, observers may not be aware of all lapses of attention [54], so using self-report as an index of attentional lapses could theoretically miss real differences in lapse prevalence across load conditions. However, we note that we saw no evidence in behavior that observers were less engaged in the one-item condition than in the two-item condition—responses were more precise than the two-item condition, and guessing was negligible.

Third, we assumed that the selectivity of active neural representations, when present, is not greater in the two-item condition than in the one-item condition. We found that selectivity in the two-item condition exceeded that expected under a switching account, which we empirically approximated using data from the one-item condition. Based on this observation, we concluded that both items are concurrently represented at least some of the time. However, this pattern could be observed if the selectivity of active neural representations was higher in the two-item condition than in the one-item condition. In this alternative account, only 1 item is actively represented at a time, but the fidelity of the represented item increases with greater memory load. This alternative seems unlikely given that all current models predict a decline in the quality of neural representations with WM load [26,27,55].

### Does alpha-band activity reflect the maintenance of spatial positions in WM?

There is substantial evidence that alpha-band activity tracks where covert spatial attention is deployed [56–59], which raises the possibility that the spatial alpha-band representations that we report here reflect sustained attention to the remembered locations. If this is the case, do the alpha-band representations of spatial position qualify as WM representations? We argue that they do (also see [43]). Here, and in past work [42,43], we have found that alpha-band activity precisely tracks the remembered spatial positions throughout the delay period. Persistent, stimulus-specific activity of this kind is widely accepted as a signature of maintenance in WM [1]. Furthermore, in visual WM tasks, delay-period posterior alpha-band power decreases with increasing memory load [60,61] and predicts individual differences in WM capacity [60]. Thus, while alpha-band activity is likely only one of an ensemble of signals that track the online storage of information in WM [62], there is ample evidence that it provides a robust signature of both WM storage and covert spatial attention, consistent with past demonstrations of strong overlap between attention and WM [39–41].

### Reconciling concurrent representations of spatial position with rhythmic sampling models of attention

At first glance, our finding that 2 spatial representations can be concurrently represented in an active state might seem inconsistent with spatial attention studies that have provided evidence for rhythmic sampling when multiple items or locations must be attended [33,34,36–38]. Here we show that there must be periods during which multiple spatial representations are active at once, ruling out a switching account in which items alternate between “on” and “off” states. While there may be sequential representations in WM that resemble the rhythmic sampling seen in spatial attention, a complete model must allow for the concurrent representation of multiple items. Thus, if the primary active representation switches from one item to the other, then this handoff must be done in such a way that both items are actively represented during the switch.

### Sequential representation within an alpha cycle?

The smallest meaningful unit of oscillatory alpha-band activity is 1 cycle (lasting approximately 100 ms), which provides an upper limit on the rate at which information encoded by alpha-band power can change. It is possible that some other form of activity (e.g., spikes or high-frequency oscillations) rapidly cycles through the items in WM, representing each item sequentially on a much faster time scale [63,64] However, if this is the case, then this rapid sequential representation must co-exist with concurrent representations of each item encoded by alpha-band power.

### Conclusions

Although persistent, stimulus-specific activity is thought to play a central role in WM [1], there has been debate regarding how many items can be represented in an active state at once [6,11,17,18]. Using a spatial encoding model, we reconstructed active stimulus-specific representations (CTFs) from the scalp distribution of alpha-band power. We found that the selectivity of spatial CTFs was lower when 2 items were stored than when 1 item was stored. However, data-driven simulations revealed that the selectivity of spatial representations in the two-item condition could not be explained by models that propose that only a single item can be maintained in an active state at once. Thus, our findings provide evidence for the concurrent storage of multiple items in an active neural state.

## Materials and methods

### Ethics statement

The study and procedures were approved by the University of Chicago Institutional Review Board (IRB; approval number: IRB15-1290) and were conducted in accordance with the Declaration of Helsinki. Participants provided informed consent prior to commencement of the study.

### Participants

Forty-one volunteers participated in the experiment for monetary compensation ($15/hour). Participants were between 18 and 35 years old, reported normal color vision and normal or corrected-to-normal visual acuity, and provided informed consent according to procedures approved by the University of Chicago IRB. The sample included both male and female participants. We excluded participants from the final sample if fewer than 450 trials per condition remained after discarding trials contaminated by recording or ocular artifacts (see “Artifact rejection”). Eight observers were excluded because too few trials remained after artifact rejection. Data collection was terminated early for 4 observers because the data were unusable due to excessive artifacts, and for 1 observer because of a fire alarm. The final sample included 28 observers with an average of 581 (SD = 75) trials for one-item trials and 596 (SD = 69) trials for two-item trials.

### Apparatus and stimuli

We tested participants in a dimly lit, electrically shielded chamber. Stimuli were generated using Matlab (MathWorks, Natick, MA) and the Psychophysics Toolbox [65,66] and were presented on a 24” LCD monitor (refresh rate: 120 Hz, resolution: 1,080 × 1,920 pixels) at a viewing distance of approximately 100 cm. Stimuli were rendered against a gray background.

### Task procedure

Participants performed a spatial delayed-estimation task in which they remembered the spatial position of 1 or 2 sample stimuli (see Fig 1A). Participants initiated each trial with a spacebar press. Each trial began with a fixation point (0.2° in diameter) presented for 500 to 800 ms. Next, a memory array that comprised 1 or 2 sample stimuli was presented for 250 ms. Each stimulus was a blue or green circle (0.2° in diameter, equated for luminance) centered 4° of visual angle from the fixation point. On one-item trials, the sample stimulus was blue or green. On two-item trials, one stimulus was blue and the other was green. Participants were instructed to remember the spatial position of the sample stimuli as precisely as possible. The angular position of each stimulus around the fixation point was sampled from 8 position bins, each spanning a 45° wedge of angular positions (bins were centered at 0°, 45°, 90°, and so forth, see Fig 2A), with jitter added to cover all 360° of possible locations to prevent categorical coding of stimulus location. On two-item trials, the position bins that each stimulus occupied were fully counterbalanced across trials for each observer (i.e., for trials in which the probed item appeared in any given position bin, the unprobed item appeared in each of the 8 position bins equally often). Thus, the position bin that one stimulus occupied was random with respect to the other, which allowed us to reconstruct spatial CTFs for each stimulus independently. When both stimuli occupied the same position bin, their exact position within the bin was constrained so that the 2 items were separated by at least 0.2° of visual angle. The memory array was followed by a 1,000-ms delay period during which only the fixation point remained on screen. Finally, after the delay period, a cursor appeared at fixation, and the fixation point turned blue or green to indicate which stimulus should be reported. Participants reported the remembered location of the probed stimulus by using a mouse to click on the perimeter of a probe ring (8° in diameter, 0.2° thick). The color of the probed item (green or blue) was pseudorandomized across trials and conditions such that each color was probed on 50% of trials for each condition. Before starting the task, participants completed a brief set of practice trials to ensure that they understood the task.

### Electrophysiology

EEG activity were recorded from 30 active Ag/AgCl electrodes mounted in an elastic cap (Brain Products actiCHamp, Munich, Germany). The International 10–20 sites we recorded from were O2, Oz, O1, P8, P4, Pz, P3, P7, CP6, CP2, CP1, CP5, T8, C4, Cz, C3, T7, FT10, FC6, FC2, FC1, FC5, FT9, F8, F4, Fz, F3, F7, FP2, and FP1. We attached 2 additional electrodes with stickers to the left and right mastoids and placed a ground electrode in the cap at position FPz. We used a right mastoid reference during data collection, and then re-referenced to the algebraic average of the left and right mastoids offline. We recorded electrooculogram (EOG) with passive Ag/AgCl electrodes, which we used to monitor for eye movements and blinks. We recorded horizontal EOG from a bipolar pair of electrodes affixed approximately 1 cm from the external canthus of each eye, and we recorded vertical EOG from a bipolar pair of electrodes affixed above and below the right eye. We filtered data online (low cutoff = 0.01 Hz, high cutoff = 80 Hz, slope from low to high cutoff = 12 dB/octave) and digitized data at 500 Hz using Brain Vision Recorder (Brain Products, Munich, German) running on a PC. We kept impedance values below 10 kΩ.

### Eye tracking

We monitored gaze position at a sampling rate of 500 Hz using a desk-mounted EyeLink 1000 Plus infrared eye-tracking camera (SR Research, Ontario, Canada) in remote mode (without a chin rest). We obtained usable eye-tracking data for 19 out of 28 participants.

### Artifact rejection

Segmented EEG data were visually inspected for artifacts (amplifier saturation, excessive muscle noise, and skin potentials), and EOGs were inspected for ocular artifacts (blinks and eye movements). We also checked the gaze data of observers with usable eye-tracking data for ocular artifacts. We discarded trials contaminated by artifacts. We discarded electrode FT9 and FT10 for all observers because we obtained poor-quality data (excessive high-frequency noise) at these sites for most observers. Data from 1 or 2 additional electrodes were discarded for 4 participants because of excessive high-frequency noise or sudden steps in voltage that occurs when an electrode is damaged. The discarded electrodes for each participant were as follows: T7; F3; CP6 and C4; and P8. For the analysis of gaze position, we further excluded trials in which the eye tracker was unable to detect the pupil, operationalized as any trial in which the horizontal or vertical gaze position was more than 15° from fixation. At most 15 trials per observer were rejected due to this reason. For most observers (15 of 19), no trials were excluded for this reason.

Removal of trials with ocular artifacts was effective. Variation in grand-averaged HEOG as a function of the remembered stimulus position was <3.6 μV for both one-item trials and <1.7 μV for two-item trials. Given that eye movements of about 1° of visual angle produce a deflection in the HEOG of approximately 16 μV [67], the residual variation in the HEOG corresponds to variations in eye position of <0.23°. Analysis of the subset of participants (19 participants) for whom we obtained usable gaze position data corroborates the HEOG data obtained from all participants. Variation in grand-average horizontal gaze position as a function of remembered stimulus position was <0.12° for one-item trials and <0.07° for two-item trials. For comparison, for these participants, the variation in the average HEOG as a function of remembered stimulus position was <3.1 μV for one-item trials and <1.6 μV for two-item trials.

### Time-frequency analysis

To calculate frequency-specific activity at each electrode, we first band-pass filtered the baselined raw EEG data using EEGLAB (“eegfilt.m” [68]). For alpha-band analyses, the data were band-pass filtered between 8 and 12 Hz. For our exploratory analysis of a broad range of frequencies, we band-pass filtered the data in 1-Hz bands from 4 to 50 Hz (i.e., 4–5 Hz, 5–6 Hz, etc.). We applied a Hilbert transform (Matlab Signal Processing Toolbox) to the band-pass–filtered data to obtain the complex analytic signal. Instantaneous power was calculated by squaring the complex magnitude of the complex analytic signal. To reduce computation time for the IEM analysis across time and frequency, we down-sampled the matrix of power values to 1 sample every 20 ms. We down-sampled power values (i.e., after filtering and applying the Hilbert transform) so that down-sampling did not affect the calculation of power.

### IEM

Following our prior work [42,43], we used an IEM [44,45] (for review, see [46]) to reconstruct spatially selective CTFs from the topographic distribution of oscillatory power across electrodes. We assumed that the power at each electrode reflects the weighted sum of 8 spatially selective channels (i.e., neuronal populations), each tuned for a different angular location (Fig 2A). We modeled the response profile of each spatial channel across angular locations as a half sinusoid raised to the 25th power:

$$R=sin{\left(0.5\theta \right)}^{25},$$

such that θ is angular location (0°–359°) and *R* is the response of the spatial channel in arbitrary units. This response profile was circularly shifted for each channel such that the peak response of each spatial channel was centered over 1 of the 8 positions corresponding to the 8 position bins (0°, 45°, 90°, etc.; see Fig 2B).

An IEM routine was applied to each time point in the alpha-band analyses and to each time-frequency point in the time-frequency analyses. We partitioned our data into independent sets of training data and test data (see the “Training and test data” section). This routine proceeded in 2 stages (training and test). In the training stage (Fig 2C), training data (*B*_*1*_) were used to estimate weights that approximate the relative contribution of the 8 spatial channels to the observed response measured at each electrode. Let *B*_*1*_ (*m* electrodes × *n*_*1*_ measurements) be the power at each electrode for each measurement in the training set, *C*_*1*_ (*k* channels × *n*_*1*_ measurements) be the predicted response of each spatial channel (determined by the basis functions, see Fig 2B) for each measurement, and *W* (*m* electrodes × *k* channels) be a weight matrix that characterizes a linear mapping from “channel space” to “electrode space.” The relationship between *B*_*1*_, *C*_*1*_, and *W* can be described by a general linear model of the form

$$B_{1}={WC}_{1}.$$

The weight matrix was obtained via least-squares estimation as follows:

$$\hat{W}=B_{1}{C_{1}}^{T}{\left(C_{1}{C_{1}}^{T}\right)}^{-1}$$

In the test stage (Fig 2D), we inverted the model to transform the observed test data *B*_*2*_ (*m* electrodes × *n*_*2*_ measurements) into estimated channel responses, *C*_*2*_ (*k* channels × *n*_*2*_ measurements), using the estimated weight matrix, $\hat{W}$, that we obtained in the training phase:

$$\hat{C_{2}}={\left({\hat{W}}^{T}\hat{W}\right)}^{-1}{\hat{W}}^{T}B_{2}$$

Each estimated channel-response function was then circularly shifted to a common center, so the center channel was the channel tuned for the position of the probed stimulus (i.e., 0° on the “Channel Offset” axes), and then averaged these shifted channel-response functions across the 8 position bins to obtain a CTF. Finally, because the exact contributions of each spatial channel to each electrode (i.e., the channel weights, *W*) likely vary across participants, we applied the IEM routine separately for each participant, and statistical analyses were performed on the reconstructed CTFs. This approach allowed us to disregard differences in how location-selective activity is mapped to scalp-distributed patterns of power across participants and instead focus on the profile of activity in the common stimulus or “information” space [46].

### Training and test data

For the IEM procedure, we partitioned artifact-free trials into independent sets of training data and test data for each observer. Across all analyses, we partitioned the trials into 3 independent sets. We equated the number of trials for each location in each set. Because of this constraint, some excess trials were not assigned to any set. Thus, we used an iterative approach to make use of all available trials. For each iteration, we randomly partitioned the trials into sets (as just described) and performed the IEM procedure on the resulting training and test data. Therefore, the trials that were not included in any block were different for each iteration. We averaged the resulting channel-response profiles across iterations. This iterative approach reduced noise in the resulting CTFs by minimizing the influence of idiosyncrasies that were specific to any given assignment of trials to blocks. For analyses focused on alpha-band power, we performed 50 iterations. For analyses across a wide range of frequencies (which is a time-consuming procedure), we performed 10 iterations.

Once trials were assigned to the 3 sets, we averaged across trials for each stimulus location bin to obtain a matrix of power values across all electrodes for each location bin (electrodes × locations, for each time point). We used a leave-one-out cross-validation routine such that 2 of these sets served as the training data and the remaining matrix served as the test data. We applied the IEM routine using each of the 3 matrices as the test data, and the remaining 2 as the training set. The resulting CTFs were averaged across the 3 test sets. Different analyses require that the data for one- and two-item trials are partitioned into training and test sets differently depending on the goal of the analysis. In the following subsections, we outline how data were partitioned into training and test sets for each analysis.

#### Comparing CTF selectivity across conditions

In 2 analyses, we tested whether CTF selectivity varied across condition. In the first analysis, we tested whether CTF selectivity differed as a function of memory load (see Fig 3). In the second analysis, we compared CTF selectivity between the two-item condition and a condition that simulated switching using the one-item condition (see Fig 5). For these analyses, we assigned trials to training and test sets the same way. When comparing CTF properties across conditions, it is important to estimate a single encoding model that is then used to reconstruct CTFs for each condition separately. If this condition is not met, then it is difficult to interpret differences in CTF selectivity between conditions because these might result from differences between the training sets (i.e., how the model is estimated; for further discussion of this issue, see [69]). We achieved this by estimating the encoding model using a training set consisting of equal trials from each condition. Specifically, we partitioned data for each condition into 3 sets (as described above, with the additional constraint that the number of trials per location in each set was also equated across conditions). We obtained condition-neutral training data by combining data across the 2 conditions before averaging, resulting in 2 training sets that included equal numbers of trials from each condition. We then tested the model of the remaining set of data for each condition separately. Thus, we used the same training data to estimate a single encoding model and varied only the test data that were used to reconstruct CTFs for each condition.

#### Cross-training analysis

For analyses in which we assessed the similarity of multivariate patterns representing one-item and two-item trials, we again partitioned each condition into 3 sets. We then trained the IEM on 2 sets of one-item data and tested on a single set of the two-item data. For the analysis in which we plot the profile of channel responses as a function of the distance between the items, we again trained the model on 2 sets of one-item data, and tested on a single set of two-item data for each distance. Before testing the model for each distance, we first aligned the trials so that the second item was always oriented clockwise relative to the position used to test the model.

#### Time × frequency analysis

In another analysis, we tested whether the range of frequencies that carried location-specific information varied as a function of memory load (see Fig 6). For this analysis, we trained and tested the IEM within each condition separately. We trained and tested within each condition separately because we were interested in whether the frequencies that track the remembered location(s) differed between the conditions; a mixed training set would not be optimal to detect which frequencies do carry location-specific information in either condition. Thus, we partitioned data from each condition into 3 sets. As we did for the other analyses, we then trained the model using 2 out of 3 sets and tested the model using the one remaining set. Each of the 3 sets for each condition was held out as the test set. Because the IEM was trained and tested within each condition, we estimated a separate encoding model for each condition. This analysis maximizes our sensitivity to differences in the frequencies carrying spatial information between conditions. However, it is difficult to interpret any difference in CTF selectivity across conditions because these differences might result from differences in how the encoding model was estimated (see [69] for further discussion of this issue).

### Statistical analysis

#### Modeling response error

Response error was measured as the number of degrees between the presented angular location and reported angular location, such that errors ranged from 0° (a perfect response) to ±180° (a maximally imprecise response). To quantify performance, we fit a mixture model to the distribution of response errors for each participant using MemToolbox [70]. For one-item trials, we modeled the distribution of response errors as a two-component mixture model, comprising a von Mises distribution centered on the correct value (i.e., a response error of 0°), corresponding to trials in which the sample location was remembered, and a uniform distribution, corresponding to guesses in which the reported location was random with respect to the sample location. We obtained maximum likelihood estimates for 2 parameters: (1) the dispersion of the von Mises distribution (*s*.*d*.), which reflects response precision; and (2) the height of the uniform distribution (*pGuess*), which reflects the probability of guessing. For two-item trials, we fit a three-component mixture model that also included an additional von Mises component centered on the location of the unprobed item, corresponding to trials in which participants mistakenly reported the location of the unprobed item (i.e., “swaps”) [50]. We obtained maximum likelihood estimates for the same parameters as in one-item trials, with one additional parameter (*pSwap*), which reflects the probability of swaps.

#### CTF selectivity

To quantify the spatial selectivity of alpha-band CTFs, we used linear regression to estimate CTF slope. Specifically, we calculated the slope of the channel responses as a function of spatial channels after collapsing across channels that were equidistant from the channel tuned for the position of the stimulus (i.e., a channel offset of 0°). Higher CTF slope indicates greater spatial selectivity.

#### Cluster-based permutation test

We used a cluster-based permutation test to identify when CTF selectivity was reliably above chance. This procedure corrects for multiple comparisons [71,72]. To this end, we identified clusters in which CTF selectivity was greater than 0 by a performing one-sample *t* test (against 0) at each time point in the alpha-band analyses (or at each time-frequency point in the time × frequency analysis). We then identified clusters of contiguous points that exceeded a threshold of *t* = 1.703 (which corresponds to a one-sided *p-*value of 0.05 for 27 degrees of freedom). For each cluster, we calculated a test statistic by summing all *t* values in the cluster. We used a Monte Carlo randomization procedure to empirically approximate a null distribution for this test statistic. Specifically, we repeated the IEM procedure 1,000 times but randomized the position labels within each training and test set (see “Training and test data”) so that the labels were random with respect to the observed response at each electrode. For each iteration, we calculated CTF selectivity across time (or across time and frequencies) and identified clusters as described above. For each iteration, we calculated the test statistic for the largest cluster, resulting in a null distribution of 1,000 cluster test statistics. Finally, we identified clusters that had test statistics larger than the 95th percentile of the null distribution. Thus, our cluster test was a one-tailed test with an alpha level of 0.05, corrected for multiple comparisons.

### Encoding model simulations

We ran 3 simulations to test what factors influence the selectivity of CTFs reconstructed with the IEM. In our two-item condition, we isolated the CTF for the probed item from that of the unprobed item because the position of the unprobed item was independent of the probed item (i.e., the location bin that the 2 items occupied were fully counterbalanced). Because the position of the unprobed item was random relative to the probed item, the representation of the unprobed item will function as noise when reconstructing the probed item, which might interfere with the reconstruction of the probed item. In Simulation 1, we examined whether reduced CTF selectivity seen in the two-item condition could reflect an inability of our IEM procedure to accurately reconstruct the representation of interest when a second representations is present. In Simulation 2, we examined whether greater noise in the two-item condition can explain the decline in CTF selectivity with memory load. In Simulation 3, we examined whether CTF selectivity reliably reflects a true decrease in the amplitude of the spatial representation in the simulated two-item data compared to the simulated one-item data (holding noise constant between the conditions).

#### Method

For each simulation, we generated synthetic data sets that each comprised 28 “subjects” (matching the sample size in our actual experiment). Each “subject” completed 576 trials in each condition (1,152 trials total). We constrained the position of the stimuli on each trial exactly as we did in our experiment (i.e., the positions of the 2 items in the two-item condition were fully counterbalanced, and we imposed a minimum distance between the items so that they did not overlap [see “Task procedure”]). For each subject, we simulated activity across 30 electrodes, with each electrode reflecting the weighted sum of 8 spatially tuned channels. For each electrode, we randomly generated a vector of channel weights by randomly sampling 8 values (a weight for each channel) from [0, 1], which yielded a matrix of channel weights, *W* (30 electrodes × 8 channels). We modeled the response profile of these channels across spatial positions as a half sinusoid raised to the 25th power:

$$R=sin{\left(0.5\theta \right)}^{25}$$

such that θ is angular location (0°–359°) and *R* is the response of the spatial channel in arbitrary units. This response profile was circularly shifted for each channel such that the peak response of each spatial channel was centered over 1 of the 8 positions corresponding to the 8 positions bins (0°, 45°, 90°, etc.; see Fig 2A). Note that this simulated spatial tuning matched that of the basis functions used in our IEM procedure (see section “IEM”). However, we note that the outcome of our simulations do not depend on the spatial tuning of the simulated data matching that of the basis functions: we obtained the same results when we repeated the simulation described here using simulated data with broader tuning (a half sinusoid raised to the 13th power). For each trial, we determined the response of each channel given the exact position of the stimulus. For two-item trials, we determined the channel responses given the position of each item and summed these to get the final channel response (i.e., we assumed that the 2 representations were additive). This produced a matrix of channel responses across trials, *C* (8 channels × 1,152 trials). We multiplied this matrix by a matrix of random channel weights drawn from [0, 1], *W* (30 electrodes × 8 channels), to get a matrix of electrode data, *E*_*dat*_ (30 electrodes × 1,152 trials):

$$E_{dat}=WC.$$

Finally, we added Gaussian noise, *N*(0, SD) to each electrode response (SD varied across simulations, see details below), yielding a synthetic set of electrode responses for a given subject.

This procedure was repeated 28 times to generate 28 “subjects” in each sample. For each sample, we applied our IEM procedure (see section “IEM”) to each subject to reconstruct CTFs for both them one-item and two-item conditions, and calculated CTF selectivity (measured as CTF slope, see “Statistical analysis”). We used the statistical test used for our real data (a paired-samples *t* test) to test for a difference in CTF selectivity between the one-item and two-item conditions. We repeated this process many times, generating 10,000 samples to empirically approximate the sampling distribution of the mean CTF selectivity in each condition, and of the mean difference in CTF selectivity between conditions (one-item minus two-item).

#### Simulation 1

In this simulation, we simply varied the number of items (1 item versus 2 items) between the 2 conditions, keeping Gaussian noise (SD = 1) and the amplitude of the underlying channel responses equivalent between the 2 conditions. We found no evidence that CTF selectivity was different between the one-item and two-item conditions. Of our 10,000 samples, the *p*-value was less than 0.05 for 4.91% (2.73% showed 1 item > 2 items, and 2.18% showed 1 item < 2 items), as expected under the null hypothesis of no difference in CTF selectivity between conditions. S1A Fig shows sampling distributions of mean CTF selectivity for the one-item and two-item conditions (left), and for the difference in mean CTF selectivity (right). This finding establishes that CTF selectivity estimated with our IEM procedure is not affected by the presence of a second spatial representation.

#### Simulation 2

In our second simulation, we varied the noise between the 2 conditions. We added more noise to the two-item condition (SD = 2) than to the one-item condition (SD = 1). We found that mean CTF selectivity still did not reliably differ between the 2 conditions. Of our 10,000 samples, the *p*-value was less than 0.05 for 5.03% (2.64% showed 1 item > 2 items, and 2.39% showed 1 item < 2 items). However, we did find that the estimated CTF selectivity was more variable in the noise condition, such that noisier data produce noisier CTFs but do not systematically bias CTF selectivity (S1B Fig). We note that the increase in noise in the two-item condition in this simulation is substantially greater than the increase in noise due to adding a second representation alone (as in Simulation 1), which explains why we did not see more variability in the two-item condition than in the one-item condition in Simulation 1. Finally, we note that CTF selectivity was generally lower for both conditions than in Simulation 1, presumably because the training set contained more noise.

#### Simulation 3

In our third simulation, we held noise constants across the one-item and two-item conditions (SD = 1 for both conditions) but this time reduced the spatial selectivity in the two-item condition by 10% (by scaling the amplitude of the underlying channel responses, *R*, by a factor of 0.9 in the two-item condition only). If our IEM procedure accurately measures changes in the selectivity present in the data, we should observe lower CTF selectivity in the two-item condition than in the one-item condition. Indeed, we found that CTF selectivity was significantly lower (*p* < 0.05) in all 10,000 samples (S1C Fig). Thus, CTF selectivity estimated using our IEM procedure accurately reflects changes in the amplitude of spatial tuning in the data.

## Supporting information

S1 Fig **Empirically approximated sampling distribution of mean CTF selectivity for each condition (left) and for the mean difference in CTF selectivity (one-item minus two-item) (right) for Simulation 1 (A), Simulation 2 (B), and Simulation 3 (C).** Dashed lines mark the mean for each sampling distribution. Data available at https://github.com/AwhVogelLab/IEM_Sim_1vs2Items. CTF, channel-tuning function.
(TIF)

## Abbreviations

BOLD: blood oxygen level–dependent
CTF: channel-tuning function
EEG: electroencephalography
EOG: electrooculogram
fMRI: functional Magnetic Resonance Imaging
IEM: inverted encoding model
s.d.: standard deviation of von Mises distribution
SD: standard deviation
WM: working memory
