# Supplementary figures and images for: Item-specific delay activity demonstrates concurrent storage of multiple active neural representations in working memory
Source: PLoS Biol. 2019 Apr 26;17(4):e3000239. doi: 10.1371/journal.pbio.3000239 (PMC6505953; doi:10.1371/journal.pbio.3000239)

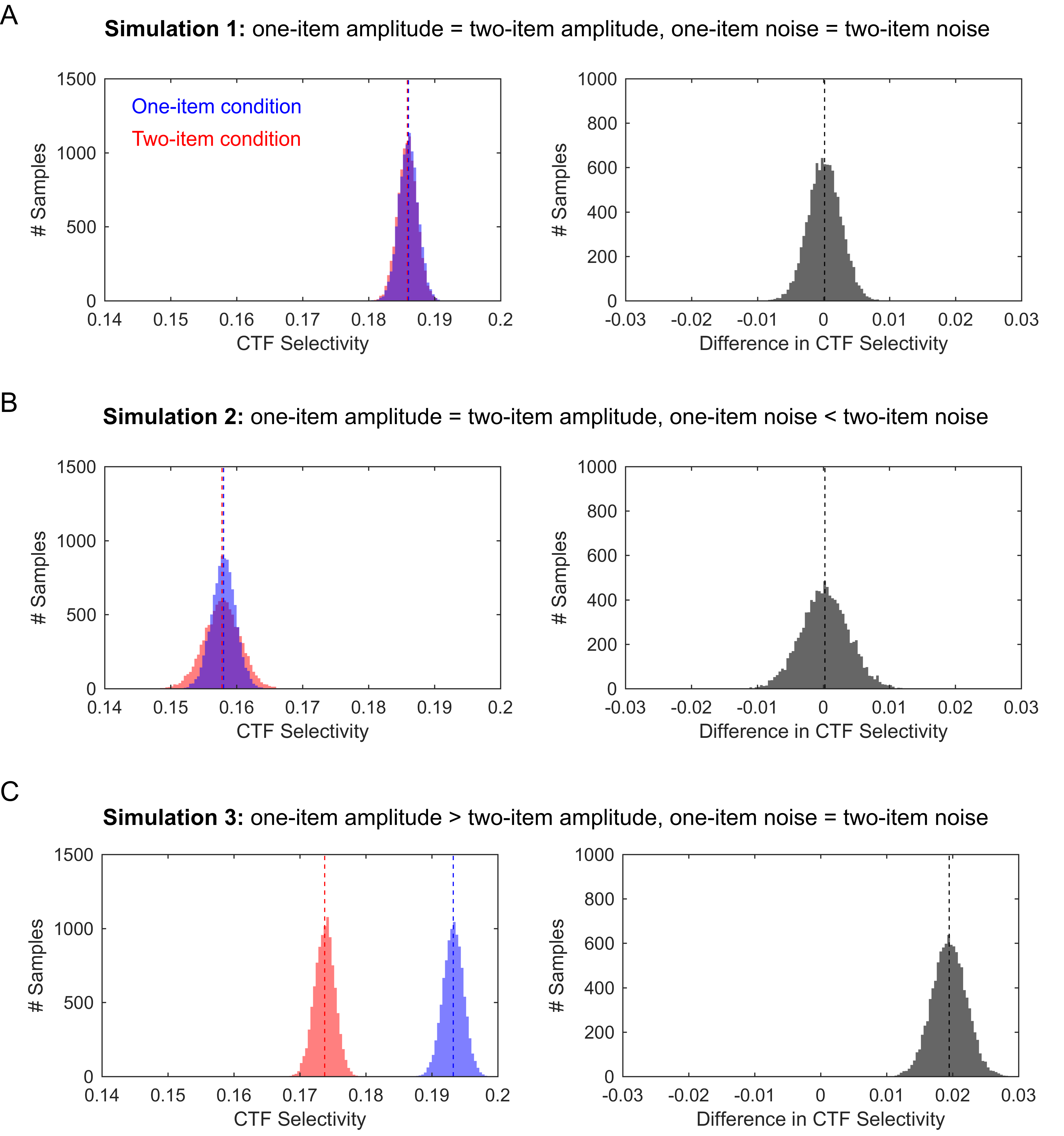

Supplement: S1 Fig — Empirically approximated sampling distribution of mean CTF selectivity for each condition (left) and for the mean difference in CTF selectivity (one-item minus two-item) (right) for Simulation 1 (A), Simulation 2 (B), and Simulation 3 (C). Dashed lines mark the mean for each sampling distribution. Data available at https://github.com/AwhVogelLab/IEM_Sim_1vs2Items. CTF, channel-tuning function. (TIF) [file pbio.3000239.s001.tif]
